# Enteric Fever in Childhood

**Published:** 1900-11-17

**Authors:** Arthur Maude

**Affiliations:** Westerham


### Hospital Clinics and Medical Progress.

### ENTERIC FEVER IN CHILDHOOD.

By Arthur Maude (Westerham).

Enteric "is not uncommon in children, but is very rare in infants" (Dreschfeld). The first half of this sentence is beyond dispute, the second more than doubtful. The figures of admissions to hospitals are no guide; the number of notifications of enteric are worthless in determining the frequency of the disease under three years old. The aberrant forms of typhoid in the young are so many, the frequency of acute febrile diarrhœa so great, that though I am not prepared to deny that enteric is rare in infants, I am not prepared to accept the statement without reserve. Even a single example of the incidence of the disease in one family is therefore of value, and such has lately been under my observation. A widower and widow (each with young children) had in their house seven children under eleven years. The mother being taken ill first, two children under three years were removed and isolated; of the others the father and one child of four alone remained uninfected; two children under fifteen months and three years were admitted suffering from enteric.

It is sought to illustrate here the essential lines of departure between the clinical aspect of the disease in children and in adults. I must be understood to treat of children under 12 years old, and to assume a familiar acquaintance with the variable phenomena of typhoid in young adults.

The early diagnosis is even more difficult in children, from the numerous febrile disturbances which present themselves in early age. Moreover the onset of most acute infectious fevers is more sudden and more pronounced in childhood, but not the onset of enteric. The initial disturbances of the nervous system, so marked a feature of acute febrile attacks in children, such as influenza, tonsillitis, pneumonia, or gastro-intestinal catarrh, are rare in typhoid. The convulsions, which so often herald any of the specific fevers of childhood, are not frequently present. Convulsions are rare as an initial symptom, though they may precede death in mortal cases, as in most infantile disorders.

It would naturally be supposed, from the frequency with which epistaxis occurs in children under many circumstances, and from the frequency of epistaxis as an initial symptom of enteric in adults, that this would be a peculiarly common early sign in children. There is no evidence, however, that it is so. It is undoubtedly of frequent occurrence, and, in contrast to older patients, it may be repeated again and again into the middle weeks of the disease.

Extreme somnolence at the onset of the disease has attracted especial remark from West, whose clinical description of enteric in childhood is now old, but is certainly not out of date. But this observation is of no real import, for many such conditions as the auto-intoxication of acute gastric catarrh, or such bacterial infections as follicular tonsillitis may produce an extreme drowsiness in children lasting several days.

The rash is an uncertain factor in children. It may be said to be either very pronounced or very unpronounced. Eustace Smith has found all rash often quite absent; Murchison, that the spots are less commonly discoverable than in adults. Rilliet and Barthez confirm this view, and add that in severe cases children have no rash. All observers agree that the typical spots are not so commonly limited to the abdomen as in grown-up patients; that they are often to be found on the back, particularly in the interscapular region, and on the loins, and are not rarely present on children's limbs. It may well be, however, that the facility with which small children can be turned for examination of the backs of their lungs may have conduced to this deduction. According to West an abundant rash is rarely seen. Thirty or forty spots scattered over the abdomen were to him quite exceptional. An abundant rash like this is only seen in severe cases, but there is no constant relation between the amount of rash and the severity of disease. Probably on the whole Dr. West (not being connected with any fever hospital) had to do with mild cases at Ormond Street. Some remarkable instances of rash have lately occurred in my practice. Four children of the same family had all highly developed spots. They were all under eleven years, very poor and ill-nourished, and the type of disease in all presented an intensity of poisoning and infectious power rarely seen in typhoid. One boy had a degree of rash quite phenomenal in abundance. On comparing it with the classical plate in Murchison's book, the spots were at least two to one. They numbered at their height hundreds, over the chest and abdomen; they were numerous on the back, arms, and thighs; they occurred on the neck, cheeks, and forehead, and even invaded the margins of the lips. This intensity of rash lasted about three days, and was succeeded for several days more by crops of a few spots. The spots were typical in every way, though a few presented the peculiarity of a larger size and more irregular oval outline than is commonly seen at other ages.

Another child, of nine, had a very copious (though not unusual) rash for two weeks; this ceased, and the temperature fell to nearly normal for two days, to be succeeded by a further rise, which was heralded for twelve hours by a fresh outcrop of spots. The whole period of rash lasted quite three weeks.

The first appearance of eruption in children is said by many writers to be earlier than in later life, occurring frequently by the fourth day. In spite of the difficulty o determining the commencement of attack in children, this is a point which my recent cases certainly confirm. Crops of spots may occur in children for a week after the temperature has reached normal.

By the older French experts sudamina were regarded as of far more common occurrence in children; Taupin found them present in 104 out of 121 cases; Louis held them as a specific indication of typhoid, an opinion few would hold now. Such highly distinguished clinicians must have been misled by the high temperatures of the ward in which the disease was handled in the middle of the nineteenth century.

The variations from a usual type of temperature chart are more frequent and more marked than in later years; the daily remission of temperature is greater; instead of a range of one degree between the maxima and minima of each day the range is more often two degrees or even more. So great are the daily declines of pyrexia during the first week that they are often subnormal, an evening reading of 102° or 103° and a morning reading of 97°. Such a condition is very misleading, and it is only by having the temperature carefully recorded at least every four hours that it is possible to arrive at an accurate diagnosis. It is rare except in hospitals that the opportunity occurs of getting accurate charts during the first week. In the first week the chart is usually very irregular, the daily rise and fall becomes more regular, and more like the type in adults during the second week, and during the period of lysis the irregularity and great daily remission again become marked. During the decline of fever it is common to find the daily remissions well below normal. The whole period of fever is shorter, and the lysis often much steeper in temperature curve than in adolescence and later life.

The duration of enteric in children is as a rule from 14 to 26 days, and the temperature may reach normal in less than a fortnight, though this is exceptional. Persistent low temperature after the decline may be recorded, especially in ill-fed, cachectic children, and it may be almost impossible to keep such children warm.

According to Eustace Smith, if any rule can be laid down as to the diurnal periodicity of temperature in children, it is that the lowest readings are obtained between 10 A.M. and noon, and the highest about midnight, thus placing the extremes several hours later than in adults. We may go further than this and say that from careful observations we often find a bidiurnal oscillation, giving a rise of temperature about 4 P.M. and another rise about midnight.

(*To be continued.*)

